# Joint Aspiration for the Diagnosis of Suspected Native Septic Arthritis

**DOI:** 10.7759/cureus.94808

**Published:** 2025-10-17

**Authors:** Juin Low, Katrina Bell, William Oliver, Andrew Duckworth

**Affiliations:** 1 Edinburgh Medical School, The University of Edinburgh, Edinburgh, GBR; 2 Department of Trauma and Orthopaedics, Royal Infirmary of Edinburgh, Edinburgh, GBR

**Keywords:** acute septic arthritis, diagnostic accuracy, joint aspiration, joint pain, surgical washout

## Abstract

Introduction: The diagnostic accuracy of these joint aspirations in suspected native septic arthritis (SA) is debated as it is unclear whether negative culture results should preclude the use of antibiotics or the need for urgent surgical intervention. This study aimed to determine the diagnostic accuracy of native joint aspirations for SA in adult patients, and to determine clinical factors associated with a positive SA diagnosis.

Methods: All adult patients (≥16 years) undergoing aspiration of a major native joint at our centre over a two-year period between September 2019 and August 2021 were identified through a retrospective search of electronic laboratory records. Data relating to patient demographics, clinical and biochemical data, joint aspiration details, post-aspiration diagnosis, management, and patient outcomes were collected and analysed. Culture-negative SA was defined using Newman’s criteria. Patients with joint prostheses or any existing orthopaedic metalwork in the affected joint were excluded.

Results: There were 161 native joint aspirations included in the study. The rate of positive diagnosis of SA was 21/161 = 13.0%, of which 16/161 = 9.9% were culture-positive SA and 5/161 = 3.1% were culture-negative SA. The presence of organisms on Gram stain analysis, a high synovial white cell count (WCC), being pyrexial, a previous diagnosis of SA, and intravenous drug use (IVDU) were associated with a diagnosis of SA (all p < 0.05). A history of crystal arthropathy made the diagnosis of SA less likely (Odds Ratio (OR) = 0.195, 95% Confidence Interval (CI) 0.044 - 0.874, p = 0.019). The diagnostic value of serum WCC and C-reactive protein (CRP) were not statistically different in the SA and non-SA groups (both p > 0.05).

Conclusion: Synovial Gram stain and culture is still the preferred diagnostic test when it comes to accurately diagnosing native joint SA. However, while culture results are pending, a high index of suspicion for SA can be based on a combination of patient’s clinical presentation, such as history of fever, previous SA, being IVDU, and a high synovial WCC. These diagnostic aids can help in guiding immediate and targeted antimicrobial therapy, and informing clinical decisions on the need for emergency surgical washout of the joint in the acute setting.

## Introduction

The acute presentation of a hot, swollen, and painful joint is a common surgical emergency and encompasses a considerably broad differential diagnosis, with the most serious time-sensitive cause being septic arthritis (SA). SA results from invasion of the synovial space by pathogenic microorganisms via haematogenous seeding or direct/contiguous spread and inoculation from adjacent tissues [1,2]. Although relatively uncommon, the sequelae of undiagnosed SA can be devastating, resulting in a high mortality rate of approximately 10% and a morbidity rate of up to 31.6% [3,4]. These include complications such as chronic pain, joint damage including osteoarthritis, reduced range of motion, osteomyelitis, systemic complications resulting in sepsis and even death. Therefore, it is crucial to diagnose SA in a timely manner to reduce morbidity and to preserve joint function because joint cartilage destruction can occur within 24 to 48 hours from initial presentation if left untreated [5].

In such patients, clinicians must maintain a high index of suspicion and be vigilant in recognising the clinical presentations of SA. As a general rule, an immediate joint aspiration for synovial fluid cell count, Gram stain, and bacterial culture and sensitivities should be performed prior to administration of antibiotics and washout [6,7]. Results of the aspiration will assist with determining the aetiology of joint effusion and subsequently to aid in early recognition and treatment. In some practice centres, the initial clinical decision of treatment is still based on the results of microscopic Gram stain analysis, particularly in favour of the relative rapidity of results being available within the hour [8]. On the other hand, cultures tend to have a longer processing time of at least 24 to 48 hours for the primary cultures to be available [8].

At present, a positive joint aspirate culture remains to be the reference standard in the diagnosis of SA [9,10]. However, the diagnostic accuracy of these joint aspirations in suspected native SA is debated as it is unclear if negative culture findings result in the need for antibiotics and for active surgical intervention. This is because Gram stain study can be unreliable in some situations and may even be influenced by previous antibiotic administration, therefore creating a false negative result [11,12]. These factors can lead to a delay in clinical decision to carry out emergency surgical washout and resulting in poorer patient outcomes. To address this knowledge gap, this study of joint aspirations was conducted with the primary aim being to establish the diagnostic accuracy of joint aspirations in diagnosing native SA using rate of positive bacterial aspirates and Newman’s criteria as the reference standard for culture-negative SA. Secondary aims were to identify clinical/comorbidity and laboratory predictors of SA and to describe one-year patient outcomes in a single institution.

This work was previously presented as a meeting abstract at the ASiT Annual Conference from 4th to 6th March 2022. The abstract was subsequently published in the British Journal of Surgery, Volume 109, Issue Supplement_6, September 2022, znac269.459, https://doi.org/10.1093/bjs/znac269.459.

## Materials and methods

Ethics approval to undertake this study was granted by the local institutional research ethics committee for a retrospective data collection of patient medical records (University of Edinburgh Research Ethics Committee issued approval reference number 2021/42). Given the anonymised retrospective cohort design of our study, with a main focus on the effect of joint aspiration results on SA, a waiver of informed patient consent was also granted by the review board.

All adult patients (aged ≥16 years) who had an aspiration of a major native joint (shoulder, elbow, wrist, hip, knee and ankle) at a large tertiary institute between September 2019 and August 2021 were identified through a retrospective search of electronic laboratory records. Data relating to patient demographics, pre-aspiration clinical data including pain, temperature, joint effusion, joint erythema, biochemical data such as white cell count (WCC), C-reactive protein (CRP), urate levels, and joint aspiration data including Gram stain microscopy (GSM), culture and sensitivity results (MC&S) were collected. Fever was defined as >38.0 °C. For joint aspiration, a positive GSM result was reported as “Presence of organisms” or “No organisms” in negative tests. Joint aspiration WCC and red cell count (RCC) were reported using ordinal categories of “Nil”, “Small”, “Moderate” and “Large” numbers. Missing data were assessed for all study variables. As the extent of missing data was minimal (<2% of the total cohort), no data imputation was performed and analyses were conducted on available cases.

Post-aspiration diagnosis, patient management (antibiotics and surgery) and patient outcomes in the SA population were collected and analysed. Exclusion criteria included patients below the age of 16 years old, if the aspiration was not from a major native joint and if there were any joint prostheses or orthopaedic metalwork present during joint aspiration.

Establishing the diagnosis of SA

The definitive diagnosis of SA remains challenging and has to be established early to prevent devastating outcomes. In our institution, all suspected SA cases had to be reviewed by senior orthopaedic surgeons. Usually, the isolation of bacteria from synovial fluid in the context of acute joint pain provides a definitive diagnosis of SA. However, the absence of organisms on Gram stain or a negative synovial fluid culture does not completely exclude the diagnosis of SA, as false negatives may sometimes occur, particularly following prior antibiotic exposure.

We adopted Newman’s criteria as the reference standard for the diagnosis of SA in the adult population [13]. According to the criteria, one of the following conditions had to be met to confirm the diagnosis: (A) isolation of a pathogenic organism from the affected joint; (B) isolation of a pathogenic organism from another source (e.g. blood source) in the context of acute joint pain suggestive of SA; (C) histological or radiological features consistent with SA; and (D) typical clinical features and turbid synovial fluid in the presence of previous antibiotic treatment [13]. In our study, Newman’s criteria C and D were utilised in cases of culture-negative SA, ensuring that patients with strong clinical evidence of SA were not excluded solely on the basis of negative cultures.

Scottish Index of Multiple Deprivation

The overall association between the diagnosis of SA and social deprivation was also examined in this study. Social deprivation was evaluated using the Scottish Index of Multiple Deprivation (SIMD) tool, which was developed by the Scottish Government in 2016 to identify and to categorise geographical areas of relative deprivation throughout Scotland [14]. The SIMD tool is derived from the patient’s postcode of residence and generally incorporates numerous components derived from social agencies, including personal income, employment status, education level, accessibility to health care services, housing development and crime rates in a specific geographical location. This tool stratifies the Scottish population into ordinal data (quintiles), where SIMD category 1 represents the most deprived and SIMD social category 5 being the least deprived.

Statistical analysis

Data were analysed using IBM SPSS software version 25.0 (IBM Corp., Armonk, NY, USA). For bivariate analysis, categorical binary data were analysed using Pearson’s chi-square test when all expected observed frequencies in each cell exceeded 5; or the Fisher’s exact test when any expected cell frequency was less than 5. To examine the differences between two ordinal or continuous data independent groups, we firstly examined the normality of distribution using the Shapiro-Wilk test, as well as kurtosis and skewness of the variable. Where ordinal or continuous data were not normally distributed, the Mann-Whitney U test was applied.

The sensitivity and specificity of the values for laboratory parameters (especially CRP) in diagnosing SA were determined by generating receiver operating characteristic (ROC) curves. An optimal cut-off value was estimated using the coordinates of the ROC curve. Statistical significance was defined as p-value of < 0.05.

## Results

Sociodemographic patient characteristics

**Figure 1:**
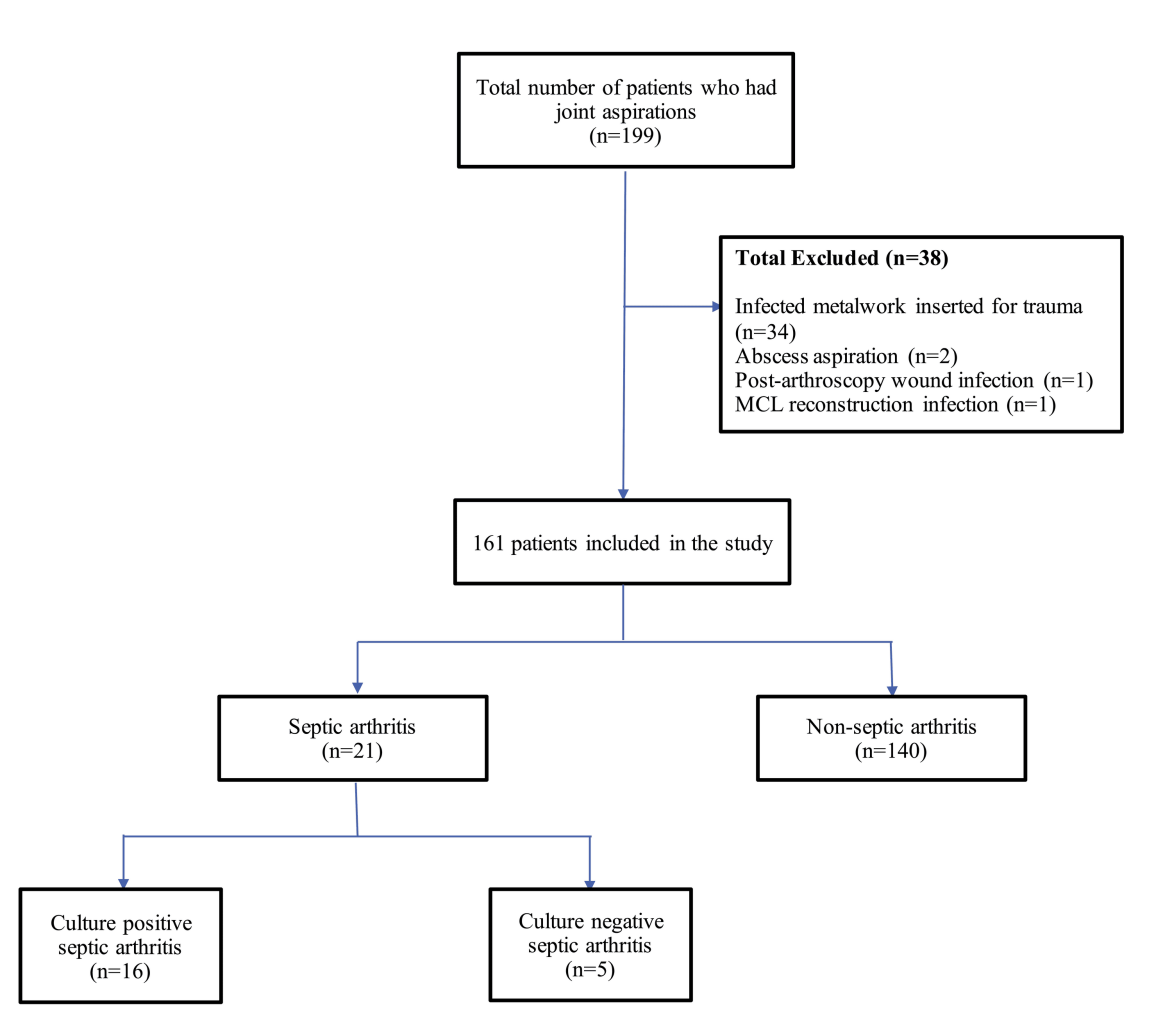
Flow diagram of patients demonstrating inclusion and exclusion criteria

After application of inclusion and exclusion criteria, 161 patients made up the study cohort (Figure 1). The mean age of aspiration of the 161 patients was 65.7 ± 16.8 years, of which 110 (68.3%) were men and 51 (31.7%) were women.

There were 21 (13.0%) native joint SA cases, of which 10 patients (47.6%) had a positive Gram stain, with the most common organism being *Staphylococcus aureus*. In the 21 native joint SA cases, 16 (76.2%) were culture-positive and five (23.8%) were culture-negative SA. In the culture-positive group, there were 13 men and three women with a mean age of 61.8 ± 17.0 years. In the culture-negative group, there were three men and two women with a mean age of 59.4 ± 24.3 years. With regard to localization of SA, the most frequently affected joints after the knee (n = 13, 61.9%) were shoulder (n = 5, 23.8%), wrist (n = 3, 14.3%), elbow (n = 1, 4.5%), and ankle (n = 1, 4.5%). Two patients presented with involvement of more than one joint, in which one had involvement of both shoulder and knee joints, and another had involvement of both wrist and ankle joints.

In the non-SA group, there were 71 crystal arthritis (50.7%), 19 inflammatory arthritis (13.6%), 19 osteoarthritis exacerbations (13.6%), eight joint haemarthrosis (0.06%), one post-viral effusion (0.01%), and 22 were classified as “others” (15.7%). The category under “others” consisted of cellulitis, bursitis, abscess or any soft tissue infections.

Newman's criteria in diagnosing septic arthritis

In our study, there were five culture-negative SA patients in which three out of five patients fulfilled Newman’s criteria C and D in the context of sterile synovial fluid (Table 1). Although Patient 1 and Patient 5 did not fulfill Newman’s criteria, it is worth noting that they were pyrexial on assessment with documented temperature of more than 38 °C, including a history of recent chest infection and history of known intravenous drug use (IVDU) which led to the diagnosis of SA. Patient 4 had reactive MRI inflammatory effusion of the glenohumeral joint, with intraosseous needle access close to the joint, which also increased the suspicion of native joint SA and was therefore treated as such.

**Table 1:** Newman’s Criteria for Diagnosis of Septic Arthritis A tick (✓) is applied to each patient fulfilling any of the criteria [13]

Patient	Criteria A	Criteria B	Criteria C	Criteria D
	Organism isolated from joint	Organism isolated from another source	No organism but presence of histological or radiological evidence of infection	No organism isolated but turbid fluid aspirated on joint aspiration and previous antibiotic use
1				
2				✓
3				✓
4			✓	
5				

Joint aspiration analysis in the SA group

The microscopy results of joint aspiration are shown in Table 2. Seven patients (33.3%) in the SA group had organisms seen on GSM and 14 (66.7%) had no organisms seen on GSM. Out of all culture-positive (n = 16) SA patients, only six (37.5%) of them had shown that there were presence of organisms on GSM. But eventually 10 (62.5%) of them grew *Staphylococcus aureus *on culture and sensitivity, which was identified as the most frequently isolated microorganism. Other organisms that were isolated on culture and sensitivity include *Streptococcus sanguinis* (n = 1, 6.3%), *Streptococcus pyogenes Group A* (n = 1, 6.3%), *Streptococcus dysgalactiae* (n = 1, 6.3%), *Neisseria gonorrhoeae* (n = 1, 6.3%), *Streptococcus agalactiae Group B* (n = 1, 6.3%), and *Micrococcus luteus* (n = 1, 6.3%).

**Table 2:** Joint Aspiration Results a : Pearson Chi Square test *: Fisher’s Exact test †: Mann-Whitney U test Dashes (-) signify that there was no data available/ not applicable Data on red cell count were unavailable for two patients, and data on white cell count were unavailable for one patient due to incomplete follow-up records. Statistically significant variables (p < 0.05) are highlighted in bold

Joint microscopy	Total (n = 161)	Septic arthritis (n = 21)	Non-septic arthritis (n = 140)	p- value	
Red cell count				0.046^†^	
Nil	34	2	32		
Small	51	6	45		
Moderate	22	2	20		
Large	52	11	41		
White cell count				0.031^†^	
Nil	7	0	7		
Small	30	2	28		
Moderate	20	1	19		
Large	103	18	85		
Presence of crystals	59	1	58	-	
Presence of organisms (Gram stain positive)	7	7	0	< 0.001*	
Turbidity of fluid	95	11	84	0.436^a^	

Joint turbidity was common but not discriminatory between the SA and non-SA groups (p = 0.436). Turbid or purulent fluid was present in nine joint aspirations (42.9%) in the culture-positive SA group, two joint aspirations (0.1%) in the culture-negative SA group and 84 (60.0%) joint aspirations in the non-SA group. Eighteen SA patients (85.7%) had large numbers of synovial WCC, as compared to 85 non-SA patients (60.7%). High synovial WCC was statistically significant in the diagnosis of SA (p = 0.031).

In evaluating the clinical characteristics (Table 3) and laboratory values (Table 4) of patients presenting with SA, joint pain (p = 1.000), joint swelling (p = 1.000), erythema (p = 1.000), hot joint (p = 0.360), and weight-bearing status (p = 1.000) were not statistically different between the two groups. Notably, the impact of social deprivation was not statistically significant between the SA and non-SA groups (p = 0.181).

**Table 3:** Clinical Predictors of Septic Arthritis α: Fisher’s Exact Test β: Pearson Chi-square test †: Mann-Whitney U test Dashes (-) signify that there was no data available/ not applicable Statistically significant variables (p < 0.05) are highlighted in bold

	Total (n = 161)	Septic arthritis (n = 21)	Non-septic arthritis (n = 140)	p- value	Odds Ratio (OR)
					(95% Confidence Interval)
Use of immunosuppressants	21	0	21	0.078^α^	0.850 (0.793 – 0.911)
Smoking	40	9	31	0.072^β^	2.484 (0.901 – 6.845)
Intravenous drug use	8	6	2	< 0.001^α^	2.286 (1.093 – 4.356)
History of diabetes mellitus	23	4	19	0.509^α^	1.486 (0.451 – 4.894)
History of rheumatoid arthritis	12	1	11	1.000^α^	0.586 (0.072 – 4.791)
History of gout/pseudogout	51	2	49	0.019^β^	0.195 (0.044 – 0.874)
Recent infection	34	7	27	0.156^α^	2.093 (0.770 – 5.687)
Previous diagnosis of septic arthritis	9	4	5	0.018^α^	6.353 (1.554 – 9.975)
Social deprivation (SIMD Quantile)				0.181^†^	1.189 (0.689 – 3.467)
1 (most deprived)	44	6	38		
2	54	5	49		
3	36	4	32		
4	16	4	12		
5 (least deprived)	11	2	9		
Clinical Signs and Symptoms					
Fever	48	11	37	0.015^β^	3.062 (1.202 – 7.801)
Joint pain	160	21	139	1.000^α^	-
Erythema	46	5	41	1.000^α^	1.030 (0.324 – 3.276)
Joint swelling	151	21	130	1.000^α^	-
Hot joint	105	16	89	0.360^α^	2.427 (0.525 – 11.228)
Ability to weight-bear	21	2	19	1.000^α^	0.784 (0.156 – 3.939)

**Table 4:** Clinical Data Values Abbreviations: IQR, Interquartile range †: Mann-Whitney U test All the clinical data values are expressed in median (IQR) Statistically significant variables (p < 0.05) are highlighted in bold

Clinical data	Septic arthritis (n = 21)	Non-septic arthritis (n = 140)	p- value
Pre-aspiration temperature (°C)	38.1 (36.9 – 38.5)	37.3 (36.6 – 38.0)	0.003^†^
Serum white cell count (10^9 ^/L)	12.9 (9.1 – 17.8)	11.3 (9.0 – 13.8)	0.143^†^
Platelet count (10^9 ^/L)	293.0 (236.0 – 405.0)	250.0 (207.0 – 320.0)	0.044^†^
Serum neutrophils (10^9 ^/L)	9.2 (6.6 – 16.1)	8.1 (6.3 – 10.4)	0.084^†^
C-reactive protein (mg/L)	197.0 (41.0 – 305.0)	89.0 (46.5 – 163.5)	0.055^†^

**Figure 2:**
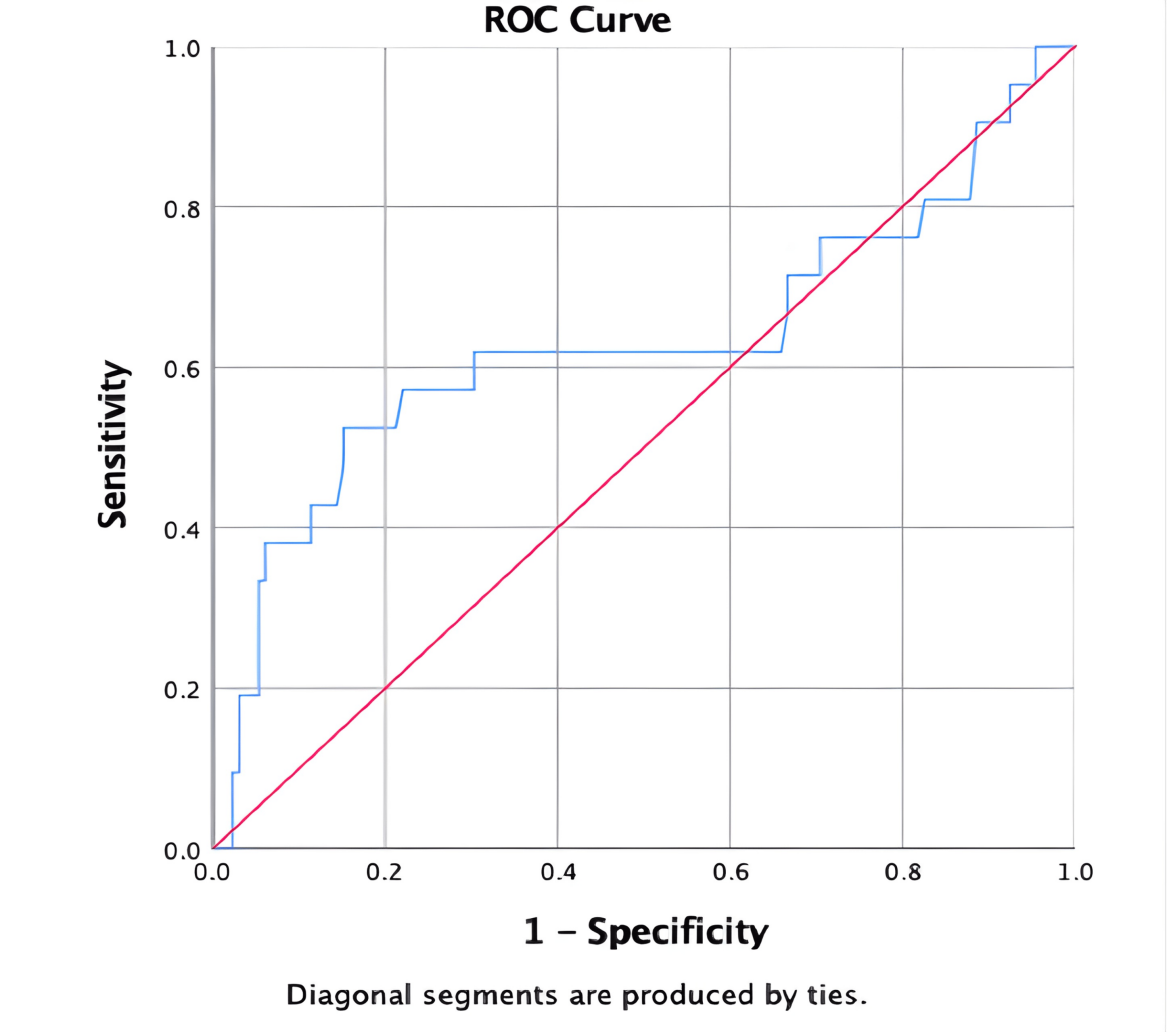
Receiver operating characteristic (ROC) curve demonstrating sensitivity and specificity of C-reactive protein (CRP) for diagnosis of native joint septic arthritis

The median serum WCC was 12.9 (10^9^ cells/L) for the SA population and 11.3 (10^9^ cells/L) for the non-SA population, which was not statistically different between the two groups (p = 0.143). The median CRP value for the SA population was 197.0 mg/L (IQR 41.0 - 305.0 mg/L) compared to the non-SA population which was 89.0 mg/L (46.5 - 163.5 mg/L) (p = 0.055). The sensitivity of CRP for SA was 61.9% and the specificity was 61.4%, using a cut-off value of ≥ 120.5mg/L. Receiver operating characteristic curve (ROC) (Figure 2) for the use of CRP to predict SA revealed an area under the curve (AUC) of 0.63 (p = 0.055).

A history of fever (p = 0.015), being IVDU (p < 0.001), and previous diagnosis of SA (p = 0.018) were statistically significant between the two groups. A history of crystal arthritis (gout/pseudogout) made the diagnosis of SA less likely (OR = 0.195, 95% CI 0.044 - 0.874) (p = 0.019).

Management and patient outcome in the SA population

The median inpatient stay for patients with proven SA was 22.8 ± 15.1 days (IQR 11 - 34 days). Supportive management in this cohort included intravenous antibiotics in all cases (n = 21, 100%), oral antibiotics in 18 patients (85.7%), arthroscopic washout in 11 patients (52.4%), and open washout in seven patients (33.3%).

From the SA cohort, recurrent presentations were observed in three patients (14.3%). All three had a course of intravenous antibiotics and underwent arthroscopic washouts. Two patients (9.5%) had recurrent SA after the initial management, presenting after being discharged 150 days and 266 days respectively. One patient with native knee SA developed concurrent osteomyelitis of the ipsilateral tibial condyle, requiring a prolonged 51-day hospital admission before self-discharging and subsequently being lost to follow-up.

The follow-up period for all 161 patients was one year. By the time of analysis, 18 patients had died, including five from the SA group. The mortality rate of SA was 14.3%. Of these, three deaths were directly attributed to SA, one had incomplete data, and one patient died of *Staphylococcus aureus* infective endocarditis, with concurrent SA not listed as the immediate cause. Death occurred at a mean of 35 days post-aspiration (range 11 - 49 days).

## Discussion

This study of joint aspirations in suspected native SA had three general aims. Primarily, to determine the diagnostic accuracy of joint aspirations in native SA by rate of positive aspirates. Secondarily, to study patient comorbidities, clinical features, laboratory parameters, and joint aspiration results in predicting SA in the adult population. Lastly, to evaluate SA patient outcomes being managed in our local institute. The most important finding of this study is that joint aspiration remains the preferred diagnostic test when it comes to accurately diagnosing native joint SA, demonstrating a high predictive value in our cohort. This study also emphasises the importance of identifying patient risk factors, utilising biochemical and joint aspiration results collectively in the comprehensive diagnostic approach of SA, rather than relying on any single test.

SA is often regarded as a diagnostic challenge owing to the heterogeneity of clinical presentations, the presence of confounding laboratory results, and the limited inaccuracy of currently available microbiologic tests. The presence of fever, IVDU, and previous diagnosis of SA had a comparable frequency in SA compared to non-SA groups. Patients who are IVDU are especially susceptible to SA due to several compounding factors. These include recurrent episodes of bacteraemia and the direct introduction of skin and environmental microorganisms via contaminated needles into the body may further predispose to mixed bacterial and fungal joint infections [15]. In our study, more than half (52.4%) of SA patients were pyrexial on assessment compared to 26.4% in the non-SA group, however, systemic features infection were less common than anticipated. Contrary to conventional medical expectation, fever - while a frequent biological response indicating presence of infection - is not a sole prerequisite for the diagnosis of SA. Several cohort studies have shown that up to 60% of patients with confirmed SA may present afebrile, particularly older adults and immunosuppressed individuals [4,16].

Our analysis has demonstrated that serum laboratory parameters for both WCC and CRP were not statistically significant between SA and non-SA, suggesting limited utility in isolation for diagnosing SA. However, it is worth mentioning that the median CRP was higher in the SA group at 197 mg/L (IQR 41.0 - 305.0 mg/L) compared to 89 mg/L (IQR 46.5 - 163.5 mg/L) in the non-SA population. Previous studies have provided mixed evidence regarding the value of CRP in diagnosing SA. A prospective study by Jeng et al. found that elevated CRP increased the likelihood (likelihood ratio 1.4) of developing SA of the hip [17], though specificity was considered mediocre in the few studies that evaluated this parameter [18,19]. A review by Margaretten et al. concluded that CRP levels did not increase the post-test probability of SA regardless of the cut-off threshold applied [20]. Similarly, Hariharan et al. conducted an analysis of 167 SA patients and reported that CRP demonstrated sensitivities of > 90% for SA, but only when low cut-offs of ≥ 20 mg/L were used [18]. Taken together, CRP, as with other inflammatory indices (WCC, ESR), should be measured as part of the work-up for suspected SA and be regarded as an adjunct to exclude the diagnosis of SA rather than being solely used as a positive predictor of SA.

In our study, a history of crystal-induced arthritis was more frequent in the non-SA group (35.0% vs. 9.5%), and its presence decreased the probability of SA (OR= 0.195, 95% CI 0.044 - 0.874) (p = 0.019). Prior cohort studies have also demonstrated that the presence of crystals, when identified in synovial fluid, is associated with a significantly reduced likelihood of the diagnosis of SA [21,22]. Of note, the presence of crystals in the synovial fluid does not conclusively exclude a septic joint and may occasionally co-exist with SA and therefore, such cases should be treated with caution. Papanicolas et al. concluded that there was concomitant SA and crystal arthropathy in 5.2% of 1612 synovial aspirates [23]. Similarly, Shah et al. reported four cases (1.5%) of concomitant positive cultures out of 265 joint aspirates containing crystals. For that reason, while the presence of crystals in joint aspiration lowers the probability of SA, it should not preclude further clinical evaluation or investigations when clinical suspicion of SA remains high.

We also evaluated the gross appearance of synovial fluid at the time of aspiration. Although turbid fluid is often regarded as suggestive of infection, our analysis demonstrated no significant association between turbidity of synovial fluid and the diagnosis of SA. We attributed that aspirated turbid synovial fluid could be non-specific and could even be subjective due to variation of fluid description reported by laboratory technicians, which remains to be an important limitation from our study on using gross appearance of synovial fluid in diagnosing SA. Another important consideration is that these turbid appearances can also occur in crystal arthropathy or other inflammatory arthritis. However, this finding contradicts Kung et al.’s retrospective study which suggested that the turbidity of the aspirated fluid was a strong positive predictor for septic hip arthritis [24]. Even so, the main difference was that their analysis involved mainly the study of both native and non-native septic hip, including the use of hip prostheses, which was part of our initial exclusion study criteria. To our knowledge, no other studies to date have directly correlated synovial fluid turbidity with a positive culture in a general patient population of native SA.

Our analysis also suggested that the presence of organisms on GSM and a high synovial WCC were highly suggestive of culture-positive SA, being consistent with the findings of other studies [24,25]. However, there is a paucity of available evidence to suggest that Gram stain is reliable in diagnosing SA. A retrospective observational study by Gbejuade et al. reported a sensitivity of only 17% for Gram stain in detecting culture-confirmed microorganisms and therefore concluded that Gram stain is unreliable in diagnosing SA [8]. Specifically for native joints, Stirling et al. found a 78% false-negative rate for Gram stain in an analysis of 143 positive synovial fluid cultures [26]. Regarding synovial WCC, several studies have shown that synovial WCC counts exceeding 50,000 cells/µL have a sensitivity of 49 - 63% and specificity of 88 - 92% for diagnosing SA [27,28]. An important limitation in our analysis is that synovial WCC was reported using ordinal categories rather than exact cellular numbers, therefore restricting our ability to objectively evaluate WCC thresholds for SA. It must not be assumed that high synovial WCC is exclusively indicative of SA, as in other inflammatory conditions, including crystal arthropathy, can also produce markedly elevated values. This variability in evidence highlights the urgent need for further larger-scale studies with the aim of developing more reliable diagnostic markers beyond the known current tests available.

Limitations

The findings of this study must be interpreted in the context of the inherent limitations. Paramount is the data quality: the retrospective nature of this study may introduce confounding factors and selection bias. Documentation of antibiotic administration prior to joint aspiration was not recorded, which precluded a complete analysis of possible effects of culture-negative SA results. The biggest limitation was that synovial WCC was reported using ordinal categories rather than exact cellular numbers which limited the ability to perform accurate and objective data analysis for the diagnostic accuracy of joint aspirations in SA. Subgroup analyses comparing clinical variables across specific non-SA categories were not performed due to small sample sizes. The discrepancy in assembled cohort size between SA and non-SA patients with a relatively small series of confirmed SA cases, raises the possibility of Type II error. Consequently, this could further reduce statistical power and robustness of adjusted comparisons, further limiting the ability to perform a meaningful multivariate analysis. In addition, laboratory data were incomplete for a small number of patients, with missing synovial red cell count values in two cases and missing white cell count data in one case due to unavailable follow-up information. However, given the minimal extent of missing data, this is unlikely to have significant influence on the interpretation of study findings. Another drawback was that this study was carried out in a single institution over a two-year period, which may occasionally limit the external validity required to support significant widespread changes in clinical practice. The greatest strength of this study, however, is the primacy of its focus: the results and methodology of this study may still be applicable to similar local institutions to determine which specific clinical factors are most predictive of SA to aid in clinician decision-making in an acute setting.

Future research directions

SA is an orthopaedic emergency, and prompt diagnosis and treatment are crucial to prevent irreversible joint damage and potentially life-threatening systemic complications. While conventional synovial WCC remains valuable and is a cornerstone of diagnostic evaluation of SA as highlighted in our results, recent studies have identified novel synovial biomarkers such as pentraxin-3 (PTX3), presepsin, and interleukin-6 (IL-6). Strong recommendations are expressed in the literature for the diagnostic accuracy of such biomarkers [29,30]. Taken together, these findings suggest that further large-scale studies are warranted to evaluate the clinical utility of these biomarkers. Integrating such markers into predictive models and synovial biochemical scoring systems could allow tailored diagnostic strategies and therefore improving confirmatory diagnosis and early management of SA.

## Conclusions

This study of joint aspirations highlights the importance of synovial fluid analysis combined with careful clinical judgment, incorporating both patient clinical factors and Newman’s criteria in the evaluation of suspected SA. Synovial Gram stain and culture remains to be the preferred diagnostic test when it comes to accurately diagnosing native joint SA. It cannot be over-emphasised: no clinical sign or laboratory test, taken solely, is conclusive for differentiating between SA and non-septic causes of acute monoarthritis, but the association of several comorbidity factors, notably a documented fever of more than 38 °C, being IVDU, previous diagnosis of SA and a high synovial WCC are positive predictors of SA and should be treated as such. Conversely, a history of crystal-induced arthritis is a negative predictor of SA. Knowledge of this information can help clinicians to assess the likelihood of SA before joint aspiration culture results are known. Further large-scale studies incorporating additional diagnostic modalities, including specific synovial fluid biomarkers such as PTX-3, presepsin and interleukins with new specific synovial fluid biochemical scores may enhance the diagnostic accuracy and timeliness of SA diagnosis.

## References

[1] Smith JW, Chalupa P, Shabaz Hasan M (2006). Infectious arthritis: clinical features, laboratory findings and treatment. Clin Microbiol Infect.

[2] Visser S, Tupper J (2009). Septic until proven otherwise: approach to and treatment of the septic joint in adult patients. Can Fam Physician.

[3] Schindler M, Huber L, Walter N (2025). Survival and risk factor analysis in patients with septic arthritis: a retrospective study of 192 cases. BMC Infect Dis.

[4] Gupta MN, Sturrock RD, Field M (2001). A prospective 2-year study of 75 patients with adult-onset septic arthritis. Rheumatology (Oxford).

[5] Momodu II, Savaliya V (2025). Septic arthritis. StatPearls.

[6] Puzzitiello RN, Lipson SE, Michaud RG Jr (2024). Effect of antibiotic administration before joint aspiration on synovial fluid white blood cell count in native joint septic arthritis. Open Forum Infect Dis.

[7] Coakley G, Mathews C, Field M (2006). BSR & BHPR, BOA, RCGP and BSAC guidelines for management of the hot swollen joint in adults. Rheumatology (Oxford).

[8] Gbejuade H, Elsakka M, Cutler L (2019). How well does synovial fluid gram staining correlate with cultures in native joint infections?. Orthop Rev (Pavia).

[9] Lenski M, Scherer MA (2014). Analysis of synovial inflammatory markers to differ infectious from gouty arthritis. Clin Biochem.

[10] Dey M, Al-Attar M, Peruffo L, Coope A, Zhao SS, Duffield S, Goodson N (2023). Assessment and diagnosis of the acute hot joint: a systematic review and meta-analysis. Rheumatology (Oxford).

[11] Zhang J, Stevenson A, Zhou AK, Khan F, Geetala R, Krkovic M (2024). The accuracy and diagnostic value of gram staining joint aspirates in suspected joint infections. Hip Int.

[12] Mathews CJ, Weston VC, Jones A, Field M, Coakley G (2010). Bacterial septic arthritis in adults. Lancet.

[13] Newman JH (1976). Review of septic arthritis throughout the antibiotic era. Ann Rheum Dis.

[14] (2006). Scottish Index of Multiple Deprivation 2006 Technical Report. Office of the Chief Statistician, Scottish Executive.

[15] Lavender TW, McCarron B (2013). Acute infections in intravenous drug users. Clin Med (Lond).

[16] Eder L, Zisman D, Rozenbaum M, Rosner I (2005). Clinical features and aetiology of septic arthritis in northern Israel. Rheumatology (Oxford).

[17] Jeng GW, Wang CR, Liu ST (1997). Measurement of synovial tumor necrosis factor-alpha in diagnosing emergency patients with bacterial arthritis. Am J Emerg Med.

[18] Hariharan P, Kabrhel C (2011). Sensitivity of erythrocyte sedimentation rate and C-reactive protein for the exclusion of septic arthritis in emergency department patients. J Emerg Med.

[19] Kaandorp CJ, Dinant HJ, van de Laar MA, Moens HJ, Prins AP, Dijkmans BA (1997). Incidence and sources of native and prosthetic joint infection: a community based prospective survey. Ann Rheum Dis.

[20] Margaretten ME, Kohlwes J, Moore D, Bent S (2007). Does this adult patient have septic arthritis?. JAMA.

[21] Couderc M, Pereira B, Mathieu S (2015). Predictive value of the usual clinical signs and laboratory tests in the diagnosis of septic arthritis. CJEM.

[22] Shah K, Spear J, Nathanson LA, McCauley J, Edlow JA (2007). Does the presence of crystal arthritis rule out septic arthritis?. J Emerg Med.

[23] Papanicolas LE, Hakendorf P, Gordon DL (2012). Concomitant septic arthritis in crystal monoarthritis. J Rheumatol.

[24] Kung JW, Yablon C, Huang ES, Hennessey H, Wu JS (2012). Clinical and radiologic predictive factors of septic hip arthritis. AJR Am J Roentgenol.

[25] Borzio R, Mulchandani N, Pivec R, Kapadia BH, Leven D, Harwin SF, Urban WP (2016). Predictors of septic arthritis in the adult population. Orthopedics.

[26] Stirling P, Faroug R, Amanat S, Ahmed A, Armstrong M, Sharma P, Qamruddin A (2014). False-negative rate of gram-stain microscopy for diagnosis of septic arthritis: suggestions for improvement. Int J Microbiol.

[27] Khan FY, Abu-Khattab M, Baagar K (2013). Characteristics of patients with definite septic arthritis at Hamad General Hospital, Qatar: a hospital-based study from 2006 to 2011. Clin Rheumatol.

[28] Carpenter CR, Schuur JD, Everett WW, Pines JM (2011). Evidence-based diagnostics: adult septic arthritis. Acad Emerg Med.

[29] Park HJ, Jeon JH, Song J (2025). Enhanced diagnostic accuracy for septic arthritis through multivariate analysis of serum and synovial biomarkers. J Clin Med.

[30] Imagama T, Seki K, Seki T (2021). Synovial fluid presepsin as a novel biomarker for the rapid differential diagnosis of native joint septic arthritis from crystal arthritis. Int J Infect Dis.

